# Diagnosis and management of postoperative wound infections in the head and neck region

**DOI:** 10.18632/oncoscience.589

**Published:** 2023-10-04

**Authors:** Filip Barbarewicz, Kai-Olaf Henkel, Florian Dudde

**Affiliations:** ^1^Department of Oral and Maxillofacial Surgery, Army Hospital Hamburg, Hamburg 22049, Germany

**Keywords:** surgical site infection, head and neck surgery

## INTRODUCTION

In everyday clinical practice at a department for oral and maxillofacial surgery, a large number of surgical procedures in the head and neck region take place under both outpatient and inpatient conditions. The basis of every surgical intervention is the patient’s consent to the respective procedure [1].

Particular attention is drawn to the general and operation-specific risks [2]. Particularly in the case of soft tissue procedures in the facial region, bleeding, secondary bleeding, scarring and infection of the surgical area are among the most common complications/risks, depending on the respective procedure [3, 4].

In order to minimize the wound infections/surgical site infections, aseptic operating conditions with maximum sterility are required [5, 6]. Furthermore, depending on the extent of the surgical procedure and the patient‘s previous illnesses, peri- and/or postoperative antibiotics should be considered in order to avoid postoperative surgical site infection [7].

Abscesses, cellulitis, phlegmone and (depending on the location of the procedure) empyema are among the most common postoperative infections in the respective surgical area [3, 4, 8]. The main pathogens of these infections are staphylococci, although mixed (germ) patterns are also possible [9].

Risk factors for the development of a postoperative surgical site infection include, in particular, increased age, smoking, multiple comorbidities and/or systemic diseases (e.g., diabetes mellitus type II) as well as congenital and/or acquired immune deficiency [10, 11].

These, as well as a number of other risk factors, should be recorded as part of the anamnesis of all patients undergoing surgical procedures, particularly in the head and neck region.

The majority of postoperative wound infections usually occur within the first postoperative days to weeks, as we were able to impressively demonstrate in a recent publication [12]. As a result, it can be concluded, as also described in other publications, that close clinical follow-up care should be carried out in the immediate postoperative period after surgical interventions in the head and neck region [12, 13]. The typical signs of inflammation can be seen as indications of a wound infection [3, 13].

Infections in the head and neck region can take a wide variety of clinical courses [12]. The greatest danger for both postoperative wound/surgical site infections and odontogenic abscesses in the facial region is in particular the tendency to spread cervically with subsequent thoracic inflammation (e.g., mediastinitis) and airway compression [12, 14]. In particular, fulminant inflammatory processes in the head and neck region often show, in addition to local clinical findings, systemic involvement in the context of sepsis. Sepsis is currently defined as an acute, life-threatening organ dysfunction resulting from a dysregulated immune response to a (suspected) infection [12, 15].

Although sepsis does not show a pathognomonic key symptom, the combination of various symptoms is indicative for a systemic effect deriving from the initial local inflammation of the head and neck region. Typical accompanying symptoms include fever, lack of consciousness, impairment of the cardiovascular system (e.g., hypotension, tachycardia) and an increased respiratory rate [16].

These and other symptoms can be easily detected using the clinically established qSOFA or SOFA score and are crucial measuring instruments in clinical sepsis diagnostics [17]. This is accompanied by obvious septic laboratory chemical constellations, as we were also able to impressively describe in the publication mentioned above [12].

In particular, the kidney values (creatinine), the platelet count, the leukocytes/C-reactive protein as well as the liver values (bilirubin) show strong pathological changes in septic infections [18, 19]. Another important laboratory chemical parameter in the diagnosis of septic inflammation is procalcitonine [19].

The treatment of postoperative septic inflammatory processes, particularly in the head and neck region, requires immediate therapy to counteract the local and systemic spread of the infection. The decisive treatment pillars are, on the one hand, targeted anti-infective therapy (broad-spectrum antibiosis) and, on the other hand, surgical infection treatment, for example in the sense of an abscess drainage, usually accompanied by a surgical opening of the former operation area/stitches.

The exact extent of the infection should be estimated intraoperatively and the infected region should be drained, in addition to an incision and antiseptic lavage [20]. Depending on the specific operation (e.g., facial fracture with osteosynthesis), concomitant infection of osseous structures and introduced foreign material must be considered [21].

There is a particular risk of a prolonged chronic infection with regard to possible biofilm formation when osteosynthesis material is inserted. Therefore, it may be necessary to exchange the foreign material through (re-)osteosynthesis [21]. Furthermore, targeted therapy for sepsis often requires an intensive care setting.

In order to prevent septic wound infections after surgical procedures, as in the recently published case report described, aseptic surgical conditions and, depending on the surgical procedure and the patient-specific risk factors, perioperative antibiotic therapy are essential [12].

The majority of wound infections often manifest themselves immediately postoperatively, so close follow-up should take place in order to initiate targeted and rapid therapy for this complication after surgical (soft tissue) procedures in the head and neck region.
